# Neuropsychiatric symptoms related to agenesis of the corpus callosum. A case report

**DOI:** 10.1192/j.eurpsy.2023.1889

**Published:** 2023-07-19

**Authors:** A. Izquierdo De La Puente, P. del Sol Calderón, M. García Moreno, R. Blanco Fernandez, M. Vizcaino Da Silva

**Affiliations:** 1 Psychiatry, Hospital Universitario Puerta de Hierro de Majadahonda, Majadahonda; 2 Psychiatry, CSM San Carlos El Escorial, El Escorial, Spain

## Abstract

**Introduction:**

We present the case of a 41-year-old male patient with multiple psychiatric diagnoses, he was diagnosed with agenesis of the corpus callosum, which explains his clinical presentation.

**Objectives:**

The objective is to carry out a brief review of the symptoms associated with the agenesis of the corpus callosum.

**Methods:**

The patient has been diagnosed with ADHD, cyclothymia, depressive anxiety disorder and social phobia. He has been treated with a multitude of drugs such as antidepressants, anxiolytics, stimulants and even low-dose antipsychotics. Despite the pharmacological treatments received, as well as the therapies, the patient’s functionality has progressively worsened, to the point of restricting going out of the home or maintaining a stable job.

Biographical data were collected, including psychomotor retardation and inappropriate laughter. He showed mannerisms such as fluttering and low frustration tolerance. He was slow to respond to his name and showed little affective resonance with his sister and parents. Restrictive interests, especially with English culture, for which he later studied English philology. On the other hand, his mother explains that he had no symbolic play and that, from early childhood, he had difficulties in relationships with peers.

Due to the aforementioned clinical manifestations, the functional worsening and the examination carried out in the consultation room, it was decided to extend the study with a brain MRI, where an agenesis of the corpus callosum was observed.

**Results:**

Agenesis of the corpus callosum is a malformation of the central nervous system, which affects one in every 4000 births. It can be partial or complete, and occurs between the 7th-20th week of gestation.

Agenesis of the corpus callosum presents with a triad of symptoms:

- Reduced interhemispheric communication of sensory-motor information.
- Increased information processing time
- Difficulty in abstract thinking.

This triad causes difficulties not only cognitively, but also socially. There is difficulty in integrating and learning new verbal and visual information. Tendency to literalism, with difficulty in understanding double meanings. They also have difficulty understanding non-verbal language and reading emotions, which makes interaction with peers difficult. All these symptoms can sometimes be confused with symptoms compatible with Autism Spectrum Disorder.

**Conclusions:**

After the diagnosis and after focusing the patient’s treatment on the most limiting symptoms of his daily life, an integrated approach was initiated, not only at a pharmacological level, with the use of antidepressants and anxiolytics, but also from a psychotherapeutic point of view, working on those areas in which the patient is most dysfunctional. He was accompanied in the disability application process, as well as helped in the search for associations for adults with ASD, finding there the answer to his symptoms and difficulties.

**Disclosure of Interest:**

None Declared

